# Echinococcosis: An Economic Evaluation of a Veterinary Public Health Intervention in Rural Canada

**DOI:** 10.1371/journal.pntd.0003883

**Published:** 2015-07-02

**Authors:** Janna M. Schurer, Ellen Rafferty, Marwa Farag, Wu Zeng, Emily J. Jenkins

**Affiliations:** 1 Department of Veterinary Microbiology, University of Saskatchewan, Saskatoon, Saskatchewan, Canada; 2 School of Public Health, University of Saskatchewan, Saskatoon, Saskatchewan, Canada; 3 Schneider Institutes for Health Policy, Heller School, Brandeis University, Waltham, Massachusetts, United States of America; Texas A&M University, UNITED STATES

## Abstract

Echinococcosis is a rare but endemic condition in people in Canada, caused by a zoonotic cestode for which the source of human infection is ingestion of parasite eggs shed by canids. The objectives of this study were to identify risk factors associated with infection and to measure the cost-utility of introducing an echinococcosis prevention program in a rural area. We analyzed human case reports submitted to the Canadian Institutes for Health Information between 2002 and 2011. Over this 10 year period, there were 48 cases associated with *E*. *granulosus/E*. *canadensis*, 16 with *E*. *multilocularis*, and 251 cases of echinococcosis for which species was not identified (total 315 cases). Nationally, annual incidence of echinococcosis was 0.14 cases per 100 000 people, which is likely an underestimate due to under-diagnosis and under-reporting. Risk factors for echinococcosis included female gender, age (>65 years), and residing in one of the northern territories (Nunavut, Yukon, or Northwest Territories). The average cost of treating a case of cystic echinococcosis in Canada was $8,842 CAD. Cost-utility analysis revealed that dosing dogs with praziquantel (a cestocide) at six week intervals to control cystic echinococcosis is not currently cost-effective at a threshold of $20,000-100,000 per Quality Adjusted Life Year (QALY) gained, even in a health region with the highest incidence rate in Canada ($666,978 -755,051 per QALY gained). However, threshold analysis demonstrated that the program may become cost-saving at an echinococcosis incidence of 13-85 cases per 100,000 people and therefore, even one additional CE case in a community of 9000 people could result in the monetary benefits of the program outweighing costs.

## Author Summary

In Canada, *Echinococcus* spp. tapeworms cycle primarily among wildlife hosts. People are infected with this parasite when they accidentally consume microscopic eggs spread by canids (e.g. dogs, wolves, coyotes, and foxes), and develop larval cysts, often in the liver or lungs. Echinococcosis can be a life-threatening medical condition with long-term health consequences and can be an economic burden for infected individuals and for the public health system. We analysed national health records to measure echinococcosis incidence and risk factors in Canada, and then used this information to determine if a program that facilitated dog deworming to prevent human infection might be economically feasible. Our model suggested that treating infected individuals is currently less expensive than preventing infection, even in the highest risk regions of Canada. However, deworming dogs might be feasible in small rural communities where at least one case was identified. Furthermore, the prevention program has many add-on benefits that contribute to overall community health, but are not measured by our model.

## Introduction

Echinococcosis, also known as hydatid disease, is a potentially fatal condition caused by zoonotic cestodes of the genus *Echinococcus* [1]. Two species are endemic to Canada: *E*. *multilocularis*, which causes alveolar echinococcosis (AE), and *E*. *canadensis* (formerly known as the G8 and G10 genotypes, or the cervid/sylvatic strains of *E*. *granulosus*), which causes cystic echinococcosis (CE) [1–3]. Human cases of echinococcosis are considered rare, resulting in approximately 0.72 hospitalizations per million people per year [4]. Domestically-acquired cases are thought be almost exclusively CE (caused by *E*. *canadensis*), and appear to occur more commonly in northern latitudes (>55°), in women, and in groups of Indigenous descent [2,4–6]. Foreign-acquired cases of echinococccosis could be caused by other species not present in Canada (e.g. *E*. *granulosus* sensu strictu), and may be associated with more severe disease. Echinococcosis is under-diagnosed in people due to an often prolonged disease progression, asymptomatic or nonspecific symptoms, and the difficulty of definitive diagnosis—especially in northern areas where medical imaging services are limited [1,7,8]. It is also under-reported, as there is no formal requirement to report human cases to national public health authorities in Canada. Recent studies highlight the need to better determine the incidence and health care burden associated with human echinococcosis in Canada, especially in rural, remote, and Indigenous communities [4,9,10].

The life cycle of *E*. *canadensis* is indirect, and utilizes wild cervids such as moose (*Alces alces*), elk (*Cervus canadensis*), and caribou (*Rangifer tarandus*) as intermediate hosts. Canids such as wolves (*Canis lupus*), coyotes (*C*. *latrans*), and dogs (*C*. *familiaris*) serve as definitive hosts [11–13]. Neither intermediate hosts nor definitive hosts are thought to suffer serious adverse effects as a result of infection; however, intermediate hosts may be at higher risk of predation due to decreased pulmonary function [14,15]. In Canada, *E*. *multilocularis* utilizes canid definitive hosts (e.g. coyotes, wolves, foxes [*Vulpes* spp.], and dogs) and rodents (arvicoline and neotomine) as normal intermediate hosts [2]. In contrast to *E*. *canadensis*, intermediate hosts of *E*. *multilocularis* experience more severe adverse effects [16]. People become infected by accidentally ingesting eggs shed by definitive hosts. Dogs have been identified as high risk reservoirs for human exposure to both species of *Echinococcus*, especially in areas where dogs can access offal or scavenge rodents as a food source, and where poverty is prevalent [1,17,18].

Worldwide, echinococcosis affects 2–3 million people per year, at an estimated cost of $750 million USD [19]. In countries where livestock strains of *E*. *granulosus* are highly prevalent, this disease represents a significant economic burden to healthcare systems, as well as to animal production systems [1,20,21]. For all forms of echinococcosis, there is the possibility for recurrence and of long-term sequelae following treatment, further increasing the burden of disease [1,22]. Multiple countries have implemented programs with various strategies to control and/or eliminate CE [1]. The most effective strategy is generally believed to be treatment of dogs with praziquantel (PZQ), a cestocide effective against *Echinoccocus* spp., at six week intervals in concert with surveillance of people, dogs and livestock (1). Dog population reduction can also factor into these programs. Few studies have calculated the cost effectiveness of CE prevention programs and none have been done in Canada where the status quo is simply to treat infected people [23–25]. The goals of this paper are to 1) report the incidence of echinococcosis based on existing national datasets, and 2) determine the cost-utility of using a CE prevention strategy (6-week PZQ dog dosing) in comparison to status quo, for a high risk health region in Canada using both public pay and societal perspectives.

## Methods

### Database

We obtained case records for Canadians diagnosed with echinococcosis from the Discharge Abstract Database (DAD) and National Ambulatory Care Reporting System (NACRS) for 2002–2011 through the Canadian Institute for Health Information (CIHI). Nationally, DAD captures all acute hospital inpatient cases, including deaths, discharges and hospital transfers; while NACRS collects ambulatory cases through voluntary submissions from day surgery, outpatient clinics and emergency department visits. Cases were coded using version 10 of the International Classification of Diseases (ICD-10) coding system of the World Health Organization. DAD did not report data from one province (Quebec -QC) unless a resident was treated out of province, but did report data from all other provinces and territories (BC—British Columbia, AB—Alberta, SK—Saskatchewan, MB—Manitoba, ON—Ontario, NL—Newfoundland and Labrador, NS—Nova Scotia, NB—New Brunswick, YT—Yukon Territories, NT—Northwest Territories, NU—Nunavut). Other omissions included MB and NB data for 2002/2003 and NB data for 2003/2004 due to delays in transitioning from ICD-9 to ICD-10 coding. NACRS abstract submissions were biased towards ON, as this was the only province with mandated reporting during the study period. Due to the small population in the 3 northern territories (YT, NT, NU), these cases were grouped together to avoid identifying patients or communities. Our dataset did differentiate between international patients and citizens, but did not report travel history or whether a person had recently immigrated to Canada. Cost and length of stay estimates were only available for 2009/2010 and 2010/2011.

Anonymized patient records from the NACRS and DAD databases were analyzed using SPSS statistical software (version 20; Chicago, Illinois, USA). Individual health card identification numbers (issued by provinces and territories to individuals enabling free access to insured health care services) were assigned a Meaningless But Unique Number (MBUN), which we used to ensure that each individual was counted only once over the study period. Treatment costs and length of stay estimates of individuals hospitalized multiple times were combined for that individual. Rural/urban and neighbourhood quintile income classifications were based on an individual’s postal code, and geographic location was reported by health region (according to a patient’s health card). Rural/urban residence categories adhered to Statistics Canada definitions: (1) Rural (outside or fringe of Census Metropolitan Areas (CMAs) or Census Agglomerations (CAs); (2) Urban Core (large urban area with ≥50 000 people for CMA or ≥10 000 people for CA); (3) Urban Fringe (small urban areas inside CMA or CA but separated from the urban core); (4) Urban areas outside CMAs/CAs (small towns with a population of 1000–10000 people and population density of ≥400 persons/km^2^) [26]. Other variables included age (categorized as <14, 15–64 and >65 years), gender, province where treatment occurred, and discharge status (i.e. a patient’s health status or anticipated location after leaving the hospital). Population proportions of infection were compared for risk factors using the Z-test, with statistical significance reported at the P<0.05 level. Only individuals over 14 years of age were included in the rural/urban comparison because the comparison dataset provided by Statistics Canada is limited to this age group. Incidence was reported as the median rate over 10 years [27].

### Economic Evaluation

For this evaluation, we focused on CE caused by *E*. *canadensis*, which is thought to be the primary species endemically transmitted in Canada [2]. We conducted a cost-utility analysis comparing a strategy for CE prevention, PZQ dog dosing, with the status quo (no prevention). Cost-utility analysis presents the cost per Quality Adjusted Life Year (QALY) and therefore captures the costs associated with CE, along with its impact on quantity and quality of life. We modelled one cohort, representing the health region with the highest incidence rate (Kelsey Trail, SK) and included residents of all ages, over the lifetime of the patient, to fully represent the long-term consequences of the disease and the possibility for recurrence. Consistent with the Canadian Agency for Drugs and Technologies in Health (CADTH) guidelines, we used a public payer perspective to represent all the public sector costs associated with CE and the PZQ dog dosing program, and thereby characterised the interests of both the prevention program and the health care system funders. Furthermore, we presented the societal perspective to represent the indirect costs, such as loss of productivity and travel expenses. We considered a cost per QALY between $20,000–100,000 as cost-effective, <$20,000 as very cost-effective and ≤$0 per QALY gained as cost-saving as per [28].

### PZQ Dog Dosing Program Description

The Kelsey Trail Health Region is home to 42 218 people (2013–2014 estimate), of which approximately half reside in population centres with veterinary services [29]. These centres also have animal control by-laws that prohibit dogs from running freely, require all owners to register their dogs annually, and impose fines on animal owners who do not remove animal waste from public areas. The PZQ dog dosing program considered in this paper included re-homing unwanted dogs from rural/remote communities, as per [30], as well as the following:

1. CE Surveillance: People and dogs monitored to identify high priority communities
2. PZQ dosing at 6 week intervals:
  - Population centres with dog bylaws and veterinary clinics—dog owners given PZQ tablets free of charge at annual registration
  - Rural/remote communities with no animal bylaws or veterinary clinics—program veterinarian injects dogs with PZQ 2–8 times annually depending on logistical constraints (e.g. accessibility, road conditions) and canine echinococcosis prevalence
3. Education: Echinococcosis teaching materials provided to primary and secondary school teachers

### Modelling

We used decision analysis to construct a Markov cohort simulation model within Treeage to determine incremental cost-utility. The model ran for 79 years or the average life expectancy of a resident of the Kelsey Trail Health Region [31], with Markov cycles occurring at one year iterations. For both the PZQ program and status quo, the model considered the transition between five CE health states (Healthy, Sick, Sequelae, Fully Recovered, Dead), each with associated costs and utilities. Transition probabilities determined the likelihood of moving between states.

### Data Inputs

Three types of costs were considered: CE treatment costs, CE indirect costs, and PZQ prevention program costs (S1 Appendix). We estimated the average treatment costs per CE case using estimates provided by CIHI and adding physician costs. Physician costs were estimated based on the Saskatchewan Ministry of Health Payment Schedule for Insured Services Provided by a Physician and expert physician opinion. PZQ program costs included a veterinarian salary [32], vehicle use for travel in and between communities, and the wholesale costs of PZQ. Finally, indirect costs included loss of production due to treatment (one month lost earnings), loss of production due to mortality (average income lost from death until life expectancy), and travel costs (car travel and hotel) [33]. All costs were provided in 2011 Canadian dollars.

We used utilities, which quantify the health wellbeing of an individual, to value the outcomes observed in each health state, with dead having a utility of zero and the healthy average Canadian having a utility of 0.93. Utility scores are weights representing preferences for different health states. The more preferred health states receive higher weights. Utilities are measured on a scale of 0–1, where 0 indicates death and 1 indicates perfect health [34]. Utility scores/weights for different health states could be obtained using Quality of Life instruments [35] and the Standard Gamble approach [36]. The sickness utility represents both the time when an individual is sick and when they were undergoing treatment. Our CE sickness utility (0.72) was based on estimates for hepatic resection and liver cancer, as these illnesses have similar treatments and outcomes [37,38]. Post treatment, those who fully recovered were assumed to return to the healthy state utility of 0.93, whereas those with sequelae had a slightly lower utility of 0.89. The sequelae utility was based on a SF-36 quality of life study of echinococcosis patients who had already undergone treatment but still experience effects of the disease [20,39].

We calculated the risk of developing CE in Kelsey Trail Health Region using the incidence of hospitalization from the CIHI databases (AE cases were excluded; Table 1). The course of disease and the likelihood of different outcomes, including the risk of recurrent echinococcosis, risk of sequelae, fatality rates and all-cause mortality rates were derived from the literature [1,22,40–42]. Costs and utilities were both discounted at a rate of 5% as recommended in the CADTH guidelines, with sensitivity analysis at 0% and 3% discount levels [43].

**Table 1:** Summary of base estimates and plausible ranges for utility and probability model inputs for cystic echinococcosis treatment and prevention in Canada.

Utility Variable	Base Estimate	Plausible Ranges	References
Utility- Healthy	0.93	1–0.86	[44]
Utility- Sickness	0.72	0.58–0.86	[37,38]
Utility- Sequelae	0.89	0.8–0.93	[20]
Utility- Dead	0	0	N/A
Cystic Echinococcosis Risk	0.000017	0.00000707–0.000033	DAD/NACRS
Relative Risk	0.19 (after 10yrs)^1^	0.4–0.05	[22]
Risk Recurrence	0.16	0.05–0.27	[1]
Risk Sequelae	0.075	0.02–0.15	[41,45]
Fatality Rate	0.03	0.01–0.05	[22,40,41]
Risk All-Cause Mortality	0.0057	0.0056–0.0059	[42]
Discount Rate (Costs & Effects)	0.05	0 & 0.03	[43]

^1^Relative Risk (RR) was calculated for each year from the start of the prevention program using a table function that decreased the RR of getting echinococcosis by 8% each year until it reached the base RR of 0.19 after 10 years.

We calculated the baseline relative risk (RR) of acquiring CE using a PZQ dosing strategy versus status quo from CE incidence estimates in Chile before and after the implementation of a similar PZQ program [22]. In the regions where the Chilean PZQ program was applied, the CE incidence in people decreased from ~60 per 100,000 to 11.8 per 100,000 within 10 years (a RR = 0.19) [22]. Therefore, to represent changes in CE risk following PZQ dosing while taking into account the time-lag between PZQ treatment and impact on human health, we used a table function that decreased the RR every year, from 0.919 the first year, until it reached the base rate of RR = 0.19 after 10 years. For the rest of the model the RR was held constant at 0.19 to represent the possibility that the program missed some infected dogs. We chose the Chilean prevention program and the associated RR as the base estimate for our study because it targeted a similar pathogen (*E*. *granulosus* sensu strictu) to that observed in Canada, as well as being one of few programs conducted on a continent rather than an island.

To ensure the validity of the model and the robustness of the findings, we conducted one-way sensitivity analyses of key variables, including risk of echinococcosis, dog-to-human ratio, fatality rate and discount rate. Plausible ranges were derived from 95% confidence intervals, inter-quartile ranges or the literature. Finally, a threshold analysis was conducted to determine the level at which PZQ dosing would be cost-saving, and to identify the minimum incidence rate that would result in a cost-effectiveness of <20,000 per QALY (Quality Adjusted Life Year).

### Ethics

This project was reviewed and approved by the University of Saskatchewan Biomedical Ethics Review Board (REB protocol number 13–51), which adheres to national standards set out by the Tri-Council for research involving humans. We report data at the level of the public health region (or pooled for the sparsely-populated northern territories) to avoid inadvertently identifying individual patients or communities.

## Results

### Statistical Analysis

Between 2002 and 2011, 384 discharge abstracts were submitted to the DAD and NACRS databases for patients under-going treatment for echinococcosis. Of these, 69 abstracts were removed from descriptive analyses either because they were duplicates (the same individual obtaining medical care on multiple occasions), or because they lacked sufficient information to be assigned an MBUN. The median annual incidence rate was 0.14 cases per 100 000 people (range: 0.12–0.25 cases per 100 000). The median age of echinococcosis cases was 46 years. The highest frequency of cases was observed in females, those aged 15–64, those residing in an urban core, and those residing in neighbourhoods with the lowest income quintile ranking (Table 2). Relative to the 2006 Census estimates of female:male ratios [46], the proportion of female cases was significantly higher than the proportion of male cases at the national level and in three provinces (BC- 13:6, P = 0.012; AB- 25:6, P = 0.032, ON- 18:11, P = 0.001). The proportion of cases in the top age category (<65 years) was significantly higher than the other two categories at the national level, and in AB, ON, and MB (P = 0.001, P<0.001, and P = 0.01, respectively). In BC the proportion of cases in the top age category was significantly higher than the proportion of cases in the youngest age category (P = 0.02), but not the middle age group.

**Table 2:** Description of patients receiving care for echinococcosis in Canada (2002–2011).

Descriptor	Frequency	% of cases^1^	Proportion of Population x 10^6^	P-value
Gender				
Female	209	66.3	13	<0.001
Male	106	33.7	7	
Age (years)				
0–14	22	7.0	4	<0.001
15–64	207	65.7	5	<0.001
≥65	86	27.3	20	Reference
Urban/Rural^2^				
Rural	29	11.2	9	0.27
Urban core	208	80.3	11	Reference
Urban fringe	5	1.9	5	0.11
Urban area outside CMA/CA	17	6.6	10	0.65
Missing	56	-		-
Neighbourhood Income Quintile^3^				
1- $14 800	91	29.3		-
2 - $25 800	65	20.9		-
3 - $35 300	59	19.0		-
4 - $46 900	50	16.1		-
5 - $78 800	46	14.8		-
Missing	4	-		-
Province^4^				
British Columbia	38	12.1	9	0.10
Alberta	51	16.2	15	0.72
Saskatchewan	17	5.4	18	0.48
Manitoba	17	5.4	15	0.97
Ontario	178	56.5	15	Reference
Quebec^5^	1	0.3	-	-
Newfoundland & Labrador	2	0.6	4	0.04
Nova Scotia	3	1.0	3	<0.01
New Brunswick	2	0.6	3	<0.01
Yukon, Northwest Territories & Nunavut	6	1.9	59	<0.001

^1^Percent of cases where descriptor data is available

^2^Only cases aged >14years used in rural/urban analysis; CMA—Census Metropolitan Area, CA—Census Agglomeration

^3^Average adjusted after-tax income for individuals calculated for 2006 in 2009 constant dollars (http://www.statcan.gc.ca/pub/75-202-x/2009000/analysis-analyses-eng.htm#a2)

^4^According to patient health card

^5^Only Quebec residents who received medical care out of province were captured in this analysis

**Fig 1:**
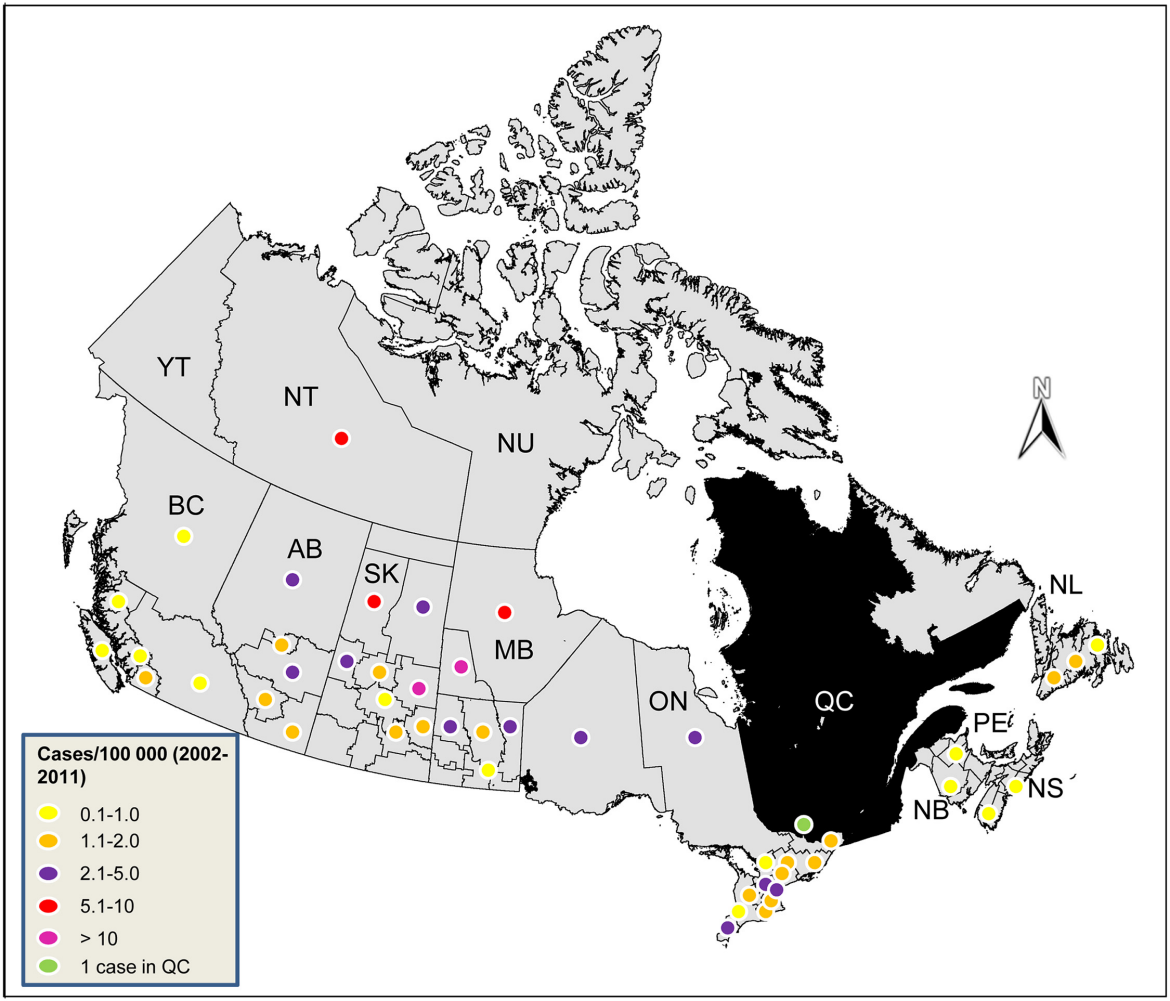
Total cumulative Echinococcosis cases per 100 000 people (2002–2011) reported by the Discharge Abstract Database and the National Ambulatory Care Reporting System Mapped by Patient Health Region (except *Quebec- QC; BC—British Columbia, AB—Alberta, SK—Saskatchewan, MB—Manitoba, ON—Ontario, NL—Newfoundland and Labrador, NS—Nova Scotia, NB—New Brunswick, YT—Yukon Territories, NT—Northwest Territories, NU—Nunavut).

We observed no significant difference in urban versus rural incidence among patients older than 14 years. The highest proportion of cases were observed in the territories (NU, NT, YK), while the lowest were observed in Atlantic Canada (NL, NS, PE, and NB). The proportion of cases in these provinces and territories were all significantly different from the proportion of cases in ON (the most populous province in Canada). Our data suggests that the majority of echinococcosis patients were treated within their province of residence (311/323, 96%), except for those residing in the territories who were all treated out of territory (NL, MB, or AB). Annual incidence rates were highest in the Kelsey Trail Health region (1.7 cases/100 000) in SK and the Norman Regional Health Authority (1.2 cases/100 000) in MB (Fig 1).

Abstracts in this dataset reported cyst location within the body in 60% of cases (188/315), and the species of *Echinococcus* in 20% of cases (64/315; Table 3). For CE, the most commonly reported cyst location was lung, followed by liver, multiple sites, and bone; whereas liver and multiples sites were the most common descriptors for AE. Of the 305 cases that described discharge disposition, 2.3% ended in fatality.

**Table 3:** Cyst location and *Echinococcus* species in Canadian echinococcosis cases (2002–2011).

ICD-10 code	Species	Cyst location	Frequency	% of cases
B67.0	*E*. *granulosus* ^1^	Liver	16	5.1
B67.1	*E*. *granulosus* ^1^	Lung	21	6.7
B67.2	*E*. *granulosus* ^1^	Bone	2	0.6
B67.3	*E*. *granulosus* ^1^	Multiple sites	8	2.5
B67.4	*E*. *granulosus* ^1^	Unspecified site	1	0.3
B67.5	*E*. *multilocularis*	Liver	8	2.5
B67.6	*E*. *multilocularis*	Multiple sites	6	1.9
B67.7	*E*. *multilocularis*	Unspecified site	2	0.6
B67.8	*Echinococcus* ^2^	Liver	141	44.8
B67.9	*Echinococcus* ^2^	Unspecified site	110	34.9

^1^ Presumably *E*. *canadensis* under new taxonomy, if domestically-acquired

^2^Species unspecified

### Economic Evaluation

We based our model on the Kelsey Trail Health Region (SK), which had the highest CE incidence rate in Canada (1.7 cases/100 000). The average cost to treat a single CE case was $8,841.68. The prevention program has a significant yearly cost, approximately $654,033, due to high numbers of dogs in rural areas and high drug costs (S1 Appendix). Furthermore the analysis showed a very small utility gain from using a prevention program compared to status quo (Incr. QALY = +0.00031870). All these factors resulted in a very high incremental cost-utility ratio (ICUR) for the base public pay case ($755,051 per QALY gained). Moreover, the societal perspective did not drastically change the outcome, with a cost per QALY gained of $666,978 (Table 4).

**Table 4:** Base case incremental cost per QALY and total costs (Can$).

Payment Perspective	Average Costs per Person ($)	Average QALY per Person	Incremental Cost-Utility ratio (ICUR)
**Public Pay**			
Status quo	8.13	17.3070	
Prevention (PZQ)	248.76	17.3073	
Incremental	+240.63	+0.0003187	$755,051 per QALY gained
**Societal**			
Status Quo	58.63	17.3070	
Prevention (PZQ)	271.20	17.3073	
Incremental	+212.56	+0.00031870	$666,978 per QALY gained

One-way sensitivity analysis demonstrated that none of the cost-utility ratios for any of the plausible variable ranges were under $100,000 per QALY. The best cost-utility ratio came at the high range of the plausible risk of CE (0.0000316 or incidence of 3.16 per 100,000) using the societal perspective with a cost per QALY of approximately $311,143. Varying other data inputs did not significantly change the outcomes, most likely because the starting incidence was so low, thereby making other probabilities irrelevant. Sensitivity of the analysis to the risk of developing CE prompted us to conduct a threshold analysis to determine at what incidence the prevention program might be considered cost-effective. This analysis found at an incidence between 10–37 cases per 100,000 the cost per QALY would be approximately $20,000, while an incidence of 13–85 per 100,000 (risk = 0.000014) would result in the program becoming cost-saving (Table 5).

**Table 5:** Threshold and sensitivity analyses for the societal perspective.

Variable	Cost per QALY (Can$)
Risk of Disease- Societal	
Low (0.00000707)	1,748,684
High (0.0000316)	311,143
Threshold (0.0001)	20,000
Threshold (0.00013)	0
Risk of Disease- Public Pay	
Low (0.00000707)	1,836,755
High (0.0000316)	399,220
Threshold (0.00037)	20,000
Threshold (0.00085)	0

## Discussion

We report an echinococcosis incidence rate of 0.14 cases per 100 000 annually, which is slightly higher than a previous Canadian estimate, likely because we included cases where echinococcosis was not the primary diagnosis. This is lower than CE incidence rates in other endemic countries including Spain, Portugal, Italy, Greece, and China; but is higher than New Zealand or the island of Tasmania which are provisionally free following the success of control programs [1,22]. We believe that our incidence rate under-estimates the true incidence because CE is not nationally notifiable to public health authorities in Canada, cases were removed from analysis due to incomplete identifier data, the dataset does not include all emergency room discharges or private clinics, and because up to 60% of CE cases (especially those caused by the sylvatic form in Canada) are thought to be asymptomatic [1]. Furthermore, over 150 cases of unspecified liver disease were reported annually during the study time period, suggesting that under-diagnosis of echinococcosis may occur [47]. Based on the best data currently available, we were not able to determine what proportion of cases were foreign-acquired; however, the universal nature of health care in Canada means that costs of treatment of foreign-acquired cases are still incurred.

Although the majority of case reports did not differentiate between CE and AE, highlighting another weakness in reporting, our findings suggest that most CE cases were likely to be domestically-acquired. The geographic distribution of CE cases (Fig 1) is very similar to the known range of *E*. *canadensis* in cervids and wolves in Canada (prevalent in the north, absent in the Atlantic provinces) [2, 12, 13], further supporting that these cases are likely endemically acquired. The highest incidence rates were in northern areas of SK, MB and the territories(YK, NU and NT) as opposed to health regions where large metropolises are present (Fig 1). All of the top primary, secondary and tertiary immigration destinations in English-speaking Canada (Toronto, Vancouver, Calgary, Edmonton, Winnipeg, Hamilton, Ottawa, Saskatoon, Victoria, Regina, and Halifax) had very low incidence rates [48]. This emphasizes the need for veterinary public health efforts and improved awareness of *Echinococcus* transmission in northwestern Canada. Sixteen individuals were diagnosed with AE over the ten year study period, which could be explained by *E*. *multilocularis* emergence or incorrect use of ICD codes by physicians. In Canada, AE cases are generally thought to be foreign-acquired, as no autochthonous cases have been reported in Canada since 1928 [49]. However, six of these individuals resided in northern health regions of BC, AB, ON, and in the northern territories (YK, NU, NT), where immigration rates are presumably low. *Echinococcus multilocularis* is has been observed in wildlife in BC, AB, SK, MB, NT, and NU, and European strains of this tapeworm were recently detected in a domestic dog (as AE) and in wild canids (as adult cestodes) [13,47,50,51]. European strains may have greater zoonotic potential than strains of the parasite long established in the southern parts of the western Canadian provinces (AB, SK, and MB), and this may be supported by the recent emergence of AE in dogs in Canada [49, 50, 52], which is more typically seen in regions of Europe highly endemic for *E*. *multilocularis*. Heightened surveillance for AE is warranted, as it generally results in worse health outcomes and significantly higher treatment costs than for CE [53].

Our findings that echinococcosis diagnosis occurred more commonly in females and older adults are comparable with other Canadian studies [4,6]. Cases of CE were most likely to have pulmonary or hepatic involvement, which is a common finding for the cervid strains in people. These findings of gender, age and tissue predilection site are risk factors shared by wildlife cervid hosts for *E*. *canadensis* [6,12,54]. The highest frequency of CE cases occurred in low income neighborhoods but we were unable to determine if the proportion of cases relative to other income quintiles was significantly different. Low income individuals might be at higher risk of CE if they fed raw offal to pets and were unable to afford regular cestocidal dosing for dogs.

We report a CE treatment cost that is similar to that in the UK ($10 215 USD), but far higher than that in other countries such as Jordan ($524 USD) [41]. At an ICUR of $755,051 per QALY gained, the dog dosing prevention program was not cost-effective relative to other funded health care programs and current willingness-to-pay guidelines [28]. The current incidence and drug costs, including indirect (societal) costs, yields an ICUR of $666,978 per QALY gained, which is also not cost-effective. To date, few CE/AE prevention programs have been evaluated from an economic perspective [53]. This is a major gap in the literature as these programs may be very cost-effective in higher incidence countries. In fact, although PZQ dosing is not currently cost-effective at the health region level, it may be cost-effective at a smaller community level. The average population per community in Kelsey Trail Health Region is 660 people; therefore, even one CE case per year in a community could warrant a prevention strategy in that community, especially if the source of CE is linked back to the dog population, rather than directly from wildlife. According to the threshold analysis, even one CE case in a community of 9000 people a year could be potentially cost saving for society. Furthermore, as CE incidence increases, the cost-effectiveness of a prevention program becomes more and more dependent on the indirect costs, especially productivity loss. A similar PZQ dog dosing program, delivered concurrently with sheep and goat vaccination, was thought to be cost-effective or even cost saving in Shiqu County (China), where the incidence of human CE (caused by *E*. *granulosus* livestock strains) and AE is extremely high [55]. Therefore, it is important that other CE/AE endemic countries engage in evaluations to determine the cost-effectiveness of echinococcosis prevention programs using domestic estimates for incidence, cost, and targeting the strains endemic in their region.

Important considerations exist that might further impact the cost-utility and feasibility of the PZQ dosing program. First, veterinarians use PZQ to treat dogs against a wide range of cestode species in addition to *E*. *canadensis*, including *Diphyllobothrium* spp., *Taenia* spp., *Dipylidium caninum*, and *Mesocestoides* spp., some of which can infect people and/or livestock. Other treatments to prevent zoonotic diseases in dogs, such as nematocides or rabies vaccination, could easily be added to the PZQ program infrastructure at a lower cost than administering all treatments separately. Second, the World Health Organization suggests that the control of echinococcosis go through multiple phases: 1) planning; 2) attack (costly and intensive control measures implemented); 3) consolidation (only high risk animals and people targeted); and 4) maintenance [1]. While the complete eradication of echinococcosis in Canada is not feasible due to wildlife reservoir hosts, there remains the possibility of future cost reductions of the prevention program. For example, after echinococcosis rates in people and animals decreased, this low risk status could be maintained through cheaper methods (e.g. education, owner-administered PZQ, screening high risk dogs). Third, our threshold analysis demonstrated that an increased incidence of echinococcosis could markedly impact the cost-utility of a prevention program. Fourth, this program would target under-served and vulnerable populations that have poorer health outcomes, and therefore, the benefit of preventing disease in these risk groups may help to reduce health inequalities. Lastly, our results indicated that all YK, NU and NT patients travelled out of territory for treatment, which can be very expensive for the health care system, as this generally involves travel by air. In NU, more than 25% of the operations budget is spent sending patients to southern referral centres to obtain care that is unavailable in the north, which drastically increases the costs of treating CE and demonstrates the benefits of a prevention program [56].

### Conclusions

Our study provides baseline human echinococcosis data that is otherwise unavailable for Canada, since there is no national reporting or targeted surveillance. Improvements to echinococcosis surveillance could include development of serological tests that are optimal for Canadian strains (e.g. *E*. *canadensis* G8 and G10), improved classification of echinococcosis by physicians (i.e. to species and/or genotype level, which can help determine pathogenicity and if cases are endemically or foreign acquired), increased awareness of echinococcosis among physicians in areas where this parasite is prevalent, and addition of this parasite to the list of nationally notifiable pathogens. Improving surveillance would allow policy-makers and governments to make informed decisions about implementing control programs, with the knowledge that increasing incidence greatly improves the cost-utility of an echinococcosis prevention program. Although PZQ dosing did not appear to be cost effective under current conditions at the level of the health region in Canada, it might still be warranted in high risk communities, especially as there are added benefits to people, pets and wildlife in controlling *Echinococcus* and other zoonotic cestodes.

## Supporting Information

S1 Appendix Summary of data inputs, base estimates, and sensitivity analyses for cystic echinococcosis control in Canada. (DOCX)
