# Supplementary material for: Echinococcosis: An Economic Evaluation of a Veterinary Public Health Intervention in Rural Canada
Source: PLoS Negl Trop Dis. 2015 Jul 2;9(7):e0003883. doi: 10.1371/journal.pntd.0003883 (PMC4489623; doi:10.1371/journal.pntd.0003883)
Supplement: S1 Appendix — (DOCX) [file pntd.0003883.s001.docx]

**S1 Appendix. Summary of data inputs, base estimates, and sensitivity analyses for cystic echinococcosis control in Canada**

| **Costs** | **Calculation Method** | **Base costs** | **Reference** |
| --- | --- | --- | --- |
| **Direct Medical Costs** | | | |
| Emergency Hospital Costs | Average patient cost in emergency care * % of patients who receive emergency care | $767.83 * 0.22 =168.92 | NACRS |
| Inpatient Hospital Costs | Average patient cost in hospital | $10693.93 * 0.78 = $8340.55 | DAD |
| Physician Costs (Emergency) | Cost of physician care per day * days in emergency * % of patients who receive emergency care | $150 * 0.31 * 0.22= 10.23 | Expert Opinion |
| Physician Costs (Inpatient) | Cost of physician care day 1 + (cost of physician subsequent days * (Length of stay in the hospital – 1)) * % of patients who receive inpatient care | ($100 + ($40 * 7.82))*0.78 = $321.98 | Expert Opinion |
| Average Treatment Cost per Case | Emergency costs + Inpatient costs + Physician costs (Emergency + Inpatient) | $168.92 + $8,340.55+ $10.23 + $321.98 = $8,841.68 |  |
| **Direct Prevention Program Costs** | | | |
| Car | Maintenance/depreciation | $2,000 per year | Expert Opinion |
| Veterinarian | Average annual salary | $70,000 per year | [32] |
| PZQ Treatment (Tablets- Municipal) | Cost of PZQ tablets per dog per year (assuming 20lbs dog) * dog-person ratio municipal *# of people in municipal areas of KT | $90.4 per dog per year * 0.06 * 19,671 = $106 695 | [2013 Associate Veterinary Purchasing Company Ltd, Municipal Records] |
| PZQ Treatment (Injectable- Rural) | Cost of PZQ injections per dog per year (assuming 20lbs dog) * dog-person ratio rural *# of people in rural areas of KT | $68.25 per dog per year * 0.24 *22,547 = $369,320 | [2013 Associate Veterinary Purchasing Company Ltd, 30] |
| Surveillance ($1 for materials + student time) | (Cost of testing samples * # of samples) + cost of summer student | ($1 * 1000 samples) + $8000 wage = $9000 per year | Cost of similar program |
| Total program costs per year | Cost of PZQ urban dogs + cost of PZQ rural dogs + cost of vet salary + car costs + surveillance costs | $2,000 + $70,000 + $106 401 + $538 560 + $9000 = $654,033 |  |
| **Indirect (Societal) Costs** | | | |
| Production Loss from Mortality (income per year) | Median wage in SK per year of life lost | $34,430 per year of life lost | [33] |
| Production loss from Treatment | Median monthly wage in SK | $34,430/24 = $1,434.58 per case | [33] |
| Travel (mileage) | Cost per km of travel * average return distance (KT-Saskatoon) | $0.42 per km* 500km = $210 per case | Expert Opinion |
| Hotel Costs | Average hotel cost per night * average length of stay | $70 * 6 nights = $300 | Expert Opinion |
